# Frost Survival Mechanism of Vegetative Buds in Temperate Trees: Deep Supercooling and Extraorgan Freezing vs. Ice Tolerance

**DOI:** 10.3389/fpls.2019.00537

**Published:** 2019-05-09

**Authors:** Gilbert Neuner, Kristina Monitzer, Dominik Kaplenig, Julia Ingruber

**Affiliations:** Unit Functional Plant Biology, Department of Botany, University of Innsbruck, Innsbruck, Austria

**Keywords:** freeze dehydration, freezing pattern, freezing resistance, ice nucleation, stem cells, supercooling, translocated ice, vegetative buds

## Abstract

In temperate climates, overwintering buds of trees are often less cold hardy than adjoining stem tissues or evergreen leaves. However, data are scarce regarding the freezing resistance (FR) of buds and the underlying functional frost survival mechanism that in case of supercooling can restrict the geographic distribution. Twigs of 37 temperate woody species were sampled in midwinter 2016 in the Austrian Inn valley. After assessment of FR, infrared-video-thermography and cryo-microscopy were used to study the freezing pattern in and around overwintering vegetative buds. Only in four species, after controlled ice nucleation in the stem (−1.6 ± 0.9°C) ice was observed to immediately invade the bud. These buds tolerated extracellular ice and were the most freezing resistant (−61.8°C mean LT_50_). In all other species (33), the buds remained supercooled and free of ice, despite a frozen stem. A structural ice barrier prevents ice penetration. Extraorgan ice masses grew in the stem and scales but in 50% of the species between premature supercooled leaves. Two types of supercooled buds were observed: in temporary supercooling buds (14 species) ice spontaneously nucleated at −20.5 ± 4,6°C. This freezing process appeared to be intracellular as it matched the bud killing temperature (−22.8°C mean LT_50_). This response rendered temporarily supercooled buds as least cold hardy. In 19 species, the buds remained persistently supercooled down to below the killing temperature without indication for the cause of damage. Although having a moderate midwinter FR of −31.6°C (LT_50_), some species within this group attained a FR similar to ice tolerant buds. The present study represents the first comprehensive overview of frost survival mechanisms of vegetative buds of temperate trees. Except for four species that were ice tolerant, the majority of buds survive in a supercooled state, remaining free of ice. In 50% of species, extraorgan ice masses harmlessly grew between premature supercooled leaves. Despite exposure to the same environmental demand, midwinter FR of buds varied intra-specifically between −17.0 and −90.0°C. Particularly, species, whose buds are killed after temporary supercooling, have a lower maximum FR, which limits their geographic distribution.

## Introduction

In temperate woody plants, vegetative buds are often less cold hardy than adjoining stem tissues and evergreen leaves (Bannister and Neuner, 2001). Additionally, buds have been reported to survive freezing by various frost survival mechanisms (Sakai and Larcher, 1987). Buds of temperate conifers have been shown to survive by deep supercooling and extraorgan freezing (Ishikawa and Sakai, 1982; Sakai, 1982), except for *Pinus* species. Buds of pines were shown to exhibit extracellular freezing (Sakai and Eiga, 1985; Quamme, 1995; Ide et al., 1998). While reproductive buds of many angiosperms exhibit deep supercooling (Quamme, 1995), their vegetative buds, based upon previous studies, have been suggested to survive freezing temperatures 10 to 15°C lower than reproductive buds, by undergoing extracellular freezing (Sakai and Larcher, 1987). Exceptions, however, have been reported (*Pyrus syriaca*: Rajashekar and Burke, 1978) and more recently added (*Acer japonicum*: Ishikawa et al., 1997; *Malus domestica*: Pramsohler and Neuner, 2013; *Alnus alnobetula*: Neuner et al., 2019).

Ice formation and concomitant extracellular freezing do not occur in the buds that exhibit extraorgan freezing. Rather intra- and extracellular water in the buds remains supercooled down to a critical freezing temperature, at which the buds are injured. While the bud tissues are supercooling, water migrates from the cells to locations that lie outside of the bud, such as the subtending stem or the bud scales. In *A. alnobetula*, ice masses can even form inside the bud between the premature leaves (Neuner et al., 2019). This process of external formation of ice masses and subsequent freeze dehydration of the bud tissues has been termed extraorgan freezing (Ishikawa and Sakai, 1982; Sakai, 1982). Three types of extraorgan freezing have been differentiated by Sakai and Larcher (1987), based on the extent of freeze dehydration and the maximum frost hardiness of the bud. *Type I* buds are very dehydration-tolerant and, under proper conditions, can dehydrate until no freezable water remains. Such buds can survive liquid nitrogen temperatures. Lateral buds of *A. japonicum* have been shown, using NMR micro imaging, to belong to *Type I* (Ishikawa et al., 1997). *Type II* buds are not fully freeze dehydration-tolerant and become frost damaged between −35 and −50°C. Apple buds could be assigned to *Type II* based on studies using infrared differential thermal analysis (IDTA) (Pramsohler and Neuner, 2013). *Type III* buds remain only partially dehydrated. Instead, freezable water remains inside the bud cells, the cells are killed when temperatures fall below the supercooling ability of the buds (−25 to −30°C), and intracellular ice formation occurs. *Type III* extraorgan freezing has been experimentally proven in the buds of several conifers and in the reproductive buds of many temperate woody angiosperms (Sakai and Larcher, 1987; Quamme, 1995). In contrast, until now only a few vegetative buds of woody angiosperm species have been classified as *Type III* (*P. syriaca*: Rajashekar and Burke, 1978; terminal buds of *A. japonicum*: Ishikawa et al., 1997). In general, experimental evidence on the frost survival mechanism of overwintering vegetative buds of woody angiosperms is scarce.

The freezing processes in and around buds can be determined using differential thermal analysis (DTA). Upon freezing of extracellular water in the stem, a high-temperature exotherm (HTE) representing the release of heat as water changes from a liquid to a solid phase (Burke et al., 1976) is recorded in buds. In supercooling buds, additionally, freezing of supercooled intracellular water is evidenced by a low-temperature exotherm (LTE). This freezing event is associated with the death of the bud cells, which was first demonstrated by Graham and Mullin (1976) in the reproductive buds of *Rhododendron*. Using DTA, the reproductive buds of several woody angiosperms were subsequently shown to survive freezing temperatures by supercooling (*Type III*, Sakai and Larcher, 1987). LTEs could not be detected, however, in the small reproductive buds in other species, hence their frost survival mechanism remained ambiguous (Sakai and Larcher, 1987). Bud size may have also limited the ability to detect LTEs in the DTA of vegetative buds of many other angiosperms listed by Sakai and Larcher (1987). These limitations can be overcome using more sensitive technologies, such as ^1^H-nuclear magnetic resonance (NMR) microscopy (Ishikawa et al., 1997; Price et al., 1997; Ide et al., 1998) or IDTA (Neuner and Kuprian, 2014; Wisniewski et al., 2015). IDTA has recently been used to study the overall freezing response, i.e., ice nucleation, propagation, and supercooling, within and around overwintering buds of *P. abies* (Kuprian et al., 2017, 2018). In contrast to NMR microscopy (Ishikawa et al., 1997), which can observe freezing phenomena at a high level of resolution, IDTA demonstrated that the buds of *P. abies* remain free of ice despite the presence of ice in the subtending stem. Ice entrance into the bud was impeded by a bowl-like ice barrier tissue that prevents ice propagation into the supercooled bud. Cryo-microscopic inspection revealed the growth of large extraorgan ice masses in the adjoining stem below (Kuprian et al., 2017). A breakdown of supercooling was observed to occur at −18°C, triggered by an intrinsic ice nucleation event inside the bud while the ice barrier remained intact. Freezing of the bud cells proceeded within seconds and matched the frost killing-temperature, and as a result, the ice formation was suggested to be intracellular.

Based on the findings regarding the frost survival mechanism of buds of *P. abies*, the objective of the present study was, after assessment of FR, to utilize IDTA and cryo-microscopy to study the freezing response in overwintering buds of other temperate woody plants, particularly vegetative buds of angiosperms, which have not been extensively examined.

We wanted to obtain a more comprehensive understanding of the functional frost survival mechanisms of overwintering buds of temperate trees by studying the freezing pattern in and around vegetative buds. While functional frost survival mechanisms of vegetative buds are quite well understood in conifers, they are not in angiosperms. Based on individual findings (Rajashekar and Burke, 1978; Ishikawa et al., 1997; Neuner et al., 2019), we hypothesized that the majority of vegetative angiosperm buds may not be ice tolerant. In case of supercooled buds, we aimed to determine whether, and how lethal, freezing is initiated in the supercooled bud tissue and where translocated ice masses preferentially form. Further, we hypothesized that midwinter bud FR should be related to the functional frost survival strategy of overwintering buds that may define maximum bud FR. Additionally, we expected little intra-specific variation between bud FR from trees exposed to the same environmental demand.

## Materials and Methods

### Plant Material

Buds of 37 woody plant species were investigated (Supplementary Table 1). Twigs ~40 cm in length were collected in the Inn Valley close to the city of Innsbruck, Austria. Stems were typically obtained from at least three individual plants, except in the case of ornamental species, where samples were taken from a single individual. The twigs were immediately transported to the Institute of Botany in Innsbruck. Samples collected in midwinter, from 7 Jan to 23 Feb 2016, were immediately subjected to measurements and observations or stored in a cold room at 4°C for no more than 3 days. Twigs collected between 24 Feb and 2 Mar 2016 were wrapped in wet paper towels placed inside sealed plastic bags and stored in a laboratory freezer at −5°C until they were used in the experiments. Terminal buds were used in all of the experiments. Air temperature in winter 2015/16 is shown in Supplementary Figure 1.

### Differential Thermal Analysis

Differential thermal analysis (DTA) was conducted according to Burke et al. (1976) using an improved measurement procedure detailed in Kuprian et al. (2017). Computer-controlled freezing treatments were conducted in a laboratory freezer (Profiline Taurus 0986, National Lab, Mölln, Germany). Temperature control in the freezer compartment, which was set at a constant low temperature, was regulated using internal heaters controlled by temperature measurement and control software (programmed in Lab View by O. Buchner). Continuous ventilation by a ventilator (Sunon, Sunonwealth Electronic Machine Industry Co., Kaohsiung, Taiwan) inside the freezing compartment provided uniform temperature conditions. DTA measurements of living and dead (oven-dried at 80°C for 3 d) bud samples were conducted using thermocouples (Type-T) connected to a data logger (CR10, Campbell Scientific Inc., Utah, USA). Temperatures were recorded every 10 s and stored on an external storage device (SM4M Storage Modul, Campbell Scientific Inc., Utah, USA).

Twig segments (3 cm) bearing a terminal bud were excised from collected twigs. Typically, 17 samples per species were prepared for DTA. All lateral buds were removed. The solder junction of a thermocouple was fixed to the surface of the outermost bud scales of the investigated bud with a thermally conducting, self-adhesive pad (Laird Technologies, Earth City, Missouri, USA). Additionally, a 1 cm incision was made with a razor blade on the side of the stem opposite of the bud. A length of wetted sisal yarn was inserted into this incision and fixed with a knot. The bud and surrounding pad were then wrapped in aluminum foil and inserted into wells (6 to 10 mm in diameter) that had been drilled into aluminum cylinders (diameter 10 cm, height 10 cm). Dead bud samples of the same size as the living samples were similarly prepared to use as a reference. The free ends of the sisal yarns were then bundled and placed into a small beaker filled with water containing ice nucleation active (INA) bacteria (*Pseudomonas syringae* van Hall 1902) that was itself immersed in a plastic tray filled with an ice slurry. Altogether, the aluminum cylinders with the inserted samples, the bundled sisal yarn, and the plastic tray containing the ice slurry and INA solution were then transferred into the freezing compartment of the freezer. All parts were thoroughly covered with wetted paper towels and enveloped in plastic film (food wrap). This prevented drying of the sisal yarn inside the ventilated freezing compartment. After initially precooling the samples at 5°C for 45 min, a controlled temperature program was initiated with a cooling rate of −5 K/h. Ultimate target low temperatures depended on the frost killing temperature of the investigated buds, which was between −45 and −80°C.

The recorded temperature data were transferred to a computer and the temperature differential between the living and dead buds was determined by subtraction of the temperature of the dead reference sample bud from the temperature of living buds (Microsoft Office Excel, Microsoft Corporation, Redmond, USA). The differential temperature was then plotted against the temperature of the dead sample. Freezing events in the live buds could then be observed as peaks rising from a flat base line. A high-temperature exotherm (HTE), originating from the freezing of extracellular water in the stem and bud scales (triggered by INA bacteria), and a low-temperature exotherm (LTE) that originates from lethal freezing of supercooled, intracellular water inside the bud cells, could be determined in the DTA plots.

### Infrared Differential Thermal Analysis (IDTA)

The temperature-controlled freezer employed in the DTA measurements was also used to conduct IDTA of the samples. Sample temperatures were also recorded with six thermocouples. Bud infrared images were recorded using FLIR S60 or FLIR T650sc (FLIR Systems, Oregon, USA) infrared cameras during successive lowering of the temperature by −4 K/h down to below the frost killing temperature of the investigated bud. Depending on the number of samples or the size of the investigated buds, either no close-up lens or a 25 μm or 50 μm close-up lens (FLIR Systems, Oregon, USA) was used. Infrared cameras were either used from outside the freezer where the sample could be viewed through an infrared permeable inspection window (10 x 10 x 0.5 cm glass made of zinc oxide, ZnS clear grade; Vitron, Jena-Maua, Germany) or the whole camera was put inside a thermally insulated plastic box and used inside the freezer (Neuner and Kuprian, 2014). Fifteen images per second were recorded and subsequently stored as image sequences on a PC.

One to five ca. 3 cm-long twig pieces bearing a terminal bud were cut off from random branch samples collected from the different species. These twig pieces were then sectioned longitudinally with a razor blade to obtain better visibility of the overall freezing pattern. Preliminary tests using intact and longitudinally dissected twig pieces indicated that no major changes in the freezing response occurred as a result of the sectioning (data not shown). The bark side of the sectioned twig was then fixed to a thermally conducting, self-adhesive pad (Laird Technologies, Earth City, Missouri, USA) that had been mounted on a lifting plate in order to horizontally orient the cut surface of the twig piece. Six thermocouples were mounted all around but not attached to the twig sample at a 1 mm distance from each other in order to determine the sample temperature. A small ventilator (20 × 20 mm) was positioned on the side of the sample to ensure a homogenous temperature distribution on the surface of the investigated sample. The HTE was triggered by use of INA bacteria via sisal yarn (as in DTA) that was fixed to an incision in the stem opposite of the bud. The mounted bud samples were placed into a temperature-controlled freezer, and the infrared camera was focused onto the cut surface of a single twig piece when a close-up lens was employed or on the cut surface of all 15 twig pieces at once when the close-up lens was not employed.

Infrared images and video sequences were further processed using FLIR ResearchIR Max software (FLIR Systems, Oregon, USA). The thermal data of whole video sequences could be subtracted from immediately before the onset of a freezing process using this software to obtain IDTA images (Hacker and Neuner, 2007). This greatly enhanced the ability to detect where ice nucleation occurred and how it propagated. It also provided the ability to determine which, if any, tissues remained supercooled and free of ice. Relevant images were then extracted to illustrate the freezing pattern in and around buds in image plates (PowerPoint, Microsoft Corporation, Redmond, USA).

### Cryo-Microscopy

For cryo-microscopic investigation, twigs were exposed in the cooling compartment of the same temperature-controlled freezer as described above. The freezing treatment started at +5°C and was followed by a cooling rate of −3 K/h down to −10°C. The samples remained at −10°C for ~20 h before the frozen buds still inside the cooling compartment of the freezer were longitudinally dissected with a pre-cooled razor blade. For cryo-microscopic inspection, a light microscope (SZX12, Olympus Austria GmbH., Vienna, Austria) was mounted inside the same freezing compartment. By this, the dissected buds could be immediately placed on the microscope stage that had an equal temperature (−10°C) as the sample. The dissected buds could then be inspected for places where extraorgan ice masses had grown around and inside the bud and where tissues remained free of ice.

### Freezing Resistance of Buds

Freezing resistance (FR) of buds was investigated by exposing whole twigs to a set of different freezing temperatures, thus simulating low temperature stresses of various degrees. Twigs were cut to ~10 cm in length. Twigs bearing at least 10 buds were then put into sealable plastic bags (16.5 × 25 cm) that contained a layer of wet paper towels. Controlled freezing treatments were conducted inside the freezing compartment of computer-controlled laboratory freezers (Profiline Taurus 0986, National Lab, Mölln, Germany and GT 2102, Liebherr, Lienz, Austria) as described above. One control sample was maintained at 4°C throughout the experiment. After an initial settling time of 45 min at 5°C, samples were cooled at a rate of −5 K/h down to five different target temperatures (−10, −20, −30, −40, and −50°C) and held for 5 h. Subsequent warming also occurred at a rate of +5 K/h. All samples then remained under moderate illumination (40 μmol photons m^−2^s^−1^) at room temperature for 1 week. Twigs were then removed from the plastic bags and the buds were longitudinally dissected for visual inspection of frost injury. The degree of frost damage to the bud was ranked in three different classes: no damage, partial damage (50%), and total loss (100%). The percent of frost damage was then plotted against the target temperature and a logistic function (Boltzmann function) was fitted to the data using OriginPro 7G SR4 (OriginLab Corporation, Northhampton, MA, USA) software. The logistic function was used to assess LT_50_, i.e., the temperature resulting in 50% lethality of the tissue.

### Statistical Analysis

Mean values and standard error of HTE, LTE and LT_50_ of the investigated buds were calculated using IBM SPSS Statistics for Windows (Version 21.0. IBM Corp., Armonk, NY, USA). Differences between mean values of different species and bud types were tested with a one-way analysis of variance (ANOVA) and a subsequent Duncan *post hoc* test, homoscedasticity provided. In the case of a negative Levene test, the Mann–Whitney *U*-test was used as a non-parametric *post hoc* test. All analyses were conducted at a significance level of *p* ≤ 0.05.

## Results

**Figure 1:**
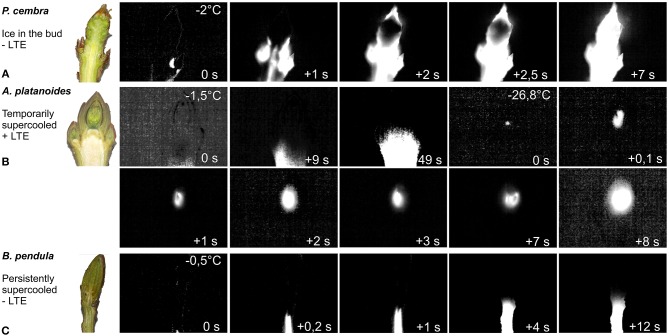
Digital color image (left) of longitudinally sectioned shoots bearing vegetative buds prior to freezing (enlarged images: Supplementary Figure 2) and IDTA images of the buds during controlled freezing at 4 K/h. Images were obtained at temperatures indicated in the upper right corner. **(A)** *P. cembra*, an example of buds that exhibit extracellular freezing (Supplementary Video 1). **(B)** *A. platanoides*: buds that exhibit supercooling. Initial ice formation occurs at −1.5°C below the bud in the adjoining stem but does not enter the bud (Supplementary Video 2). More than 6 h later, below a certain critical threshold temperature (−26.8°C), an ice nucleation event inside the bud triggers intracellular lethal freezing of all of the bud cells within 8 s (Supplementary Video 3). **(C)** *B. pendula*: buds supercool after the initial freezing of the surrounding tissue at −0.5°C, which stops below the bud (Supplementary Video 4). No further freezing process is detected, even at temperatures below the frost killing temperature. Freezing exotherm temperatures are indicated in the top right corner of each image. The time span during each freezing exotherm (in seconds) is indicated in the bottom right corner.

IDTA analysis provided the ability to directly observe the freezing pattern in and around buds, and to determine whether or not ice forms in the bud tissue. Three significantly different freezing patterns were observed. The first pattern, where ice immediately propagated from the surrounding tissue during the HTE into the bud, was observed in only four of the 37 examined species. This freezing pattern was exhibited in *Pinus cembra* buds and is shown in Figure 1A (Supplementary Video 1). This freezing pattern was also seen in *Pinus sylvestris* and two angiosperm species, *Sambucus nigra*, and *Elaeagnus rhamnoides*. In the majority (33 out of 37, or 89.2%) of the tested species, the bud tissue remained free of ice during the occurrence of the HTE (Table 1). The initial ice wave stopped below the bud, and the bud cells remained ice-free in a supercooled state. Propagation of ice from the stem into the bud was inhibited by an ice barrier in the tissue between the stem and the bud. Two types of supercooling buds were observed. In one group, a second freezing event occurred at a much lower freezing temperature than the HTE and, in addition to the infrared observations, was recorded in the DTA as a low-temperature exotherm (LTE). IDTA indicated that the LTE was localized and originated from within the bud itself. This pattern of freezing is exemplified in *Acer platanoides* (Figure 1B, Supplementary Video 2). The LTE was triggered by an ice nucleation event inside the bud in all buds that exhibited temporary supercooling. A breakdown of the ice barrier, i.e., propagation of ice from the frozen stem below a critical threshold freezing temperature, was never observed. Once ice formation was initiated in a single spot (tentatively a single cell), ice spread rapidly throughout the whole bud within seconds (Supplementary Video 3). In the second group of supercooled buds, no distinct LTE could be detected despite cooling the buds below the temperature at which the buds are killed. This freezing pattern of persistently supercooled buds, without the occurrence of a distinct LTE, is exemplified by the freezing response of *Betula pendula* (Figure 1C, Supplementary Video 4).

**Table 1:** The freezing pattern around and in buds of the 37 investigated woody species could be assigned to three different types by infrared differential thermal analysis (IDTA): *Type A* Extracellular ice formation during HTE in the bud (no ice barrier), *Type B* Temporarily supercooled and ice-free during HTE, but killed by spontaneous ice nucleation in the bud (with LTE) and *Type C* persistently supercooled and ice-free during HTE, but no ice formation down to below the frost killing temperature (no LTE).

**Bud frost survival type**	**Plant species**	**HTE ± SE (°C)**	**LTE ± SE (°C)**	**FR (±SE,°C)**	**Bud FR (°C) after others**	**Extraorgan ice masses**
*Type A*	*E. rhamnoides*	−1.4 ± 0.1	No	−41.3 ± 1.3		-
	*P. cembra*	−1.3 ± 0.1	No	−75.2 ± 4.9	−70,3^l^/−90^a^	-
	*P. sylvestris*	−1.2 ± 0.1	No	-	−70^b^/−90^a^	-
	*S. nigra*	−0.8 ± 0.1	No	-		-
*Type B*	*A. platanoides*	−1.1 ± 0.1	−23.6 ± 0.4	−32.5 ± 1.5	−40^b^	S/Sc
	*A. pseudoplatanus*	−1.2 ± 0.1	−22.4 ± 0.4	−23.8 ± 0.8	−25^b^	S/Sc^*^
	*A. hippocastanum*	−1.5 ± 0.1	−14.2 ± 0.6	−18.1 ± 0.2	−25^b^/−40^c^	Sc/L
	*C. bignoniodes*	−1.9 ± 0.2	−24.8 ± 0.2	-		L
	*C. occidentalis*	−3.2 ± 0.3	−21.6 ± 0.7	−25.0 ± 0.0		-
	*C. mas*	−1.5 ± 0.1	−22.3 ± 0.6	−24.0 ± 1.0	−30^b^	L
	*C. monogyna*	−0.7 ± 0.1	−17.7 ± 0.5	−17.0 ± 0.0		S/L
	*F. sylvatica*	−0.5 ± 0.1	−15.1 ± 0.4	−22.2 ± 2.3	−27^b^/−29.6^d^/−40^h^	Sc
	*I. aquifolium*	−1.3 ± 0.2	−29.8 ± 0.4	−28.0 ± 0.6	−18^b^	S/Sc/L
	*J. regia*	−2.1 ± 0.1	−15.9 ± 0.3	−17.7 ± 0.9	−18.5^e^	S
	*L. tulipifera*	−3.1 ± 0.2	−20.1 ± 0.1	−23.0 ± 0.6	−25^b^/−30^**b**^	S/Sc
	*P. abies*	−1.7 ± 0.2	−24.8 ± 0.2	−25.5 ± 1.1	−24,2^j^/−40^b^/−50^k^	S
	*P. x hispanica*	−2.7 ± 0.2	−17.5 ± 0.2	−21.5 ± 0.6	−30^b^	L
	*R. ferrugineum*	−1.4 ± 0.1	−15.3 ± 0.4	−17.7 ± 0.9	−25^c^	L
*Type C*	*A. incana*	−1.5 ± 0.1	No	−28.8 ± 1.3		L
	*A. alnobetula*	−1.2 ± 0.1	No	−42.5 ± 7.6	−50^g^/−45^i^	L
	*B. pendula*	−1.4 ± 0.2	No	−90.0 ± 3.3	−70^b^/−86.7^n^/−93.3^n^	L
	*C. betulus*	−1.0 ± 0.1	No	−25.7 ± 0.8	<-74^m^	L
	*C. sativa*	−3.0 ± 0.2	No	−22.0 ± 0.8	−27^f^	nd
	*C. avellana*	−1.0 ± 0.1	No	−23.7 ± 1.2	−31.9^m^	Sc
	*E. europaeus*	−1.4 ± 0.2	No	−19.0 ± 1.0		L
	*L. anagyroides*	−2.4 ± 0.3	No	−47.0 ± 0.4		nd
	*M. nigra*	−1.5 ± 0.2	No	−35.0 ± 0.0	−28.0^m^	L
	*O. carpinifolia*	−2.6 ± 0.2	No	−29.6 ± 3.0	−40^f^	L
	*P. tremula*	−1.1 ± 0.1	No	−32.0 ± 2.5	−42.4^m^	Sc/L
	*P. avium*	−1.6 ± 0.2	No	−20.0 ± 0.0	−30^o^	L
	*Q. rubra*	−1.3 ± 0.1	No	−20.8 ± 0.2	−30^b^	nd
	*S. caprea*	−0.7 ± 0.1	No	-	<-74^m^	nd
	*S. helvetica*	−1.0 ± 0.1	No	−48.1 ± 3.8	−59.9^m^	L^**^
	*S. aucuparia*	−2.8 ± 0.1	No	−32.2 ± 3.9	−40.4^m^	Sc
	*T. cordata*	−1.0 ± 0.1	No	−42.1 ± 1.7	<-70^b^	L
	*U. glabra*	−1.2 ± 0.1	No	−25.8 ± 0.8	<-70^b^	-
	*V. lantana*	−1.2 ± 0.1	No	−39.4 ± 0.7		S/L

*Species are grouped accordingly. The table lists the species examined, the mean temperature (°C) of the high temperature exotherm (HTE) and the low temperature exotherm (LTE) as determined by differential thermal analysis (DTA) and mean midwinter FR (LT_50_) of winter 2015/16, and the midwinter FR reported by other authors. For supercooling buds, locations of formation of extraorgan ice masses are additionally shown: S, subtending stem; Sc, inside bud scales; L, between premature leaves; nd, not detectable; –, not examined*.

a Bannister and Neuner (2001);

b Sakai (1982);

c Sakai and Larcher (1987);

d Hofmann et al. (2015);

e Charrier et al. (2013);

f Filippi (1986),

g Benowicz et al. (2000);

h Lenz et al. (2016);

i Neuner et al. (2019);

j Kuprian et al. (2017);

k Beuker et al. (1998);

l Buchner and Neuner (2011);

m Schiffmann (2017);

n Riikonen et al. (2013);

o Vitra et al. (2017);

* Dereuddre (1979);

** *between innermost scale and leaves*.

**Figure 2:**
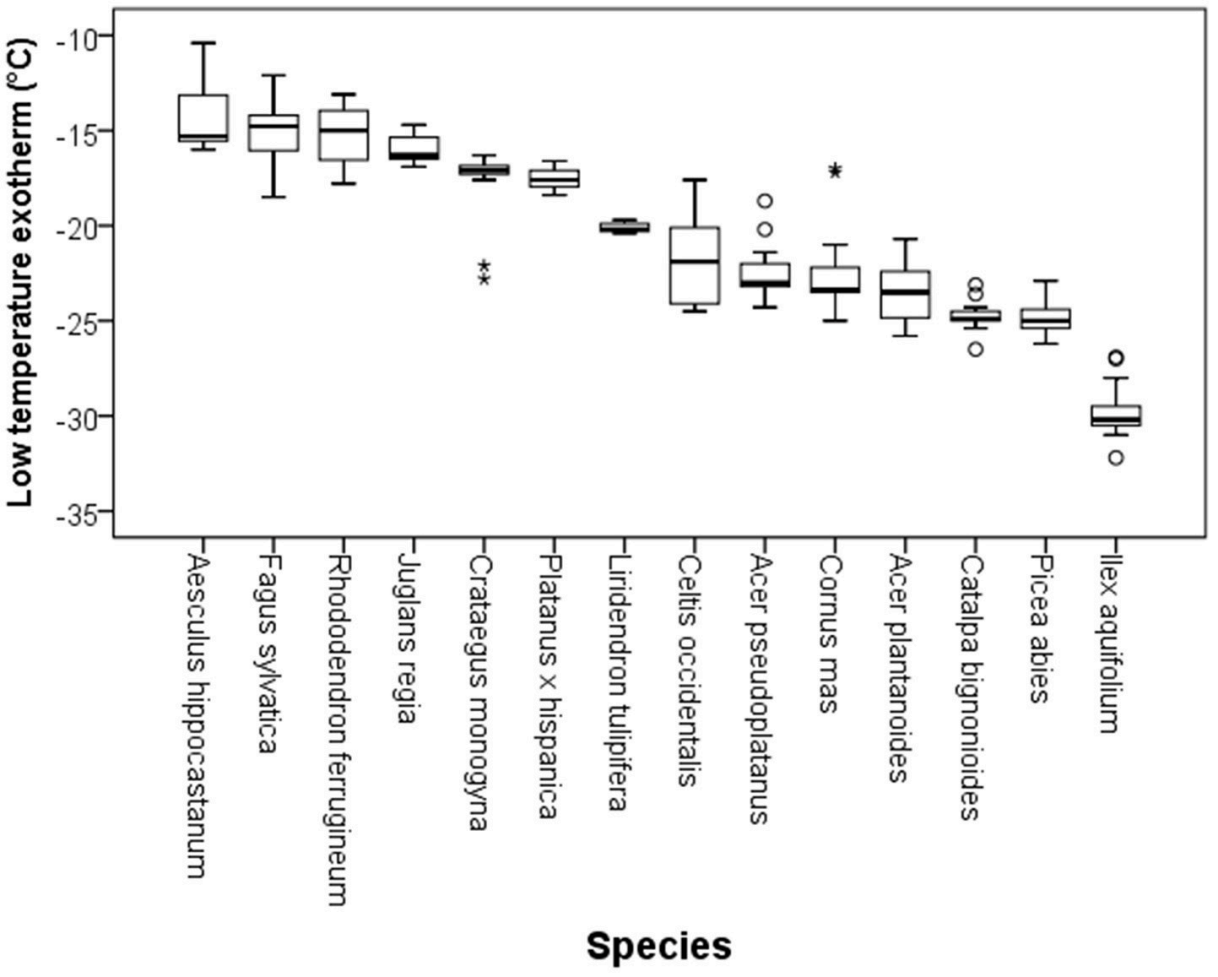
Variability in the temperatures at which low temperature exotherms (LTEs, °C) were detected by DTA in temporary supercooled buds of excised twigs of different species (*N* = 17). Ice nucleation during LTE was always initiated inside the bud. The box plots indicate the median (= second quartile; line inside the box) and extend from the first to the third quartile. The whiskers indicate at maximum the 1.5-fold interquartile range. Outliers are shown as dots, extreme outliers as stars.

**Figure 3:**
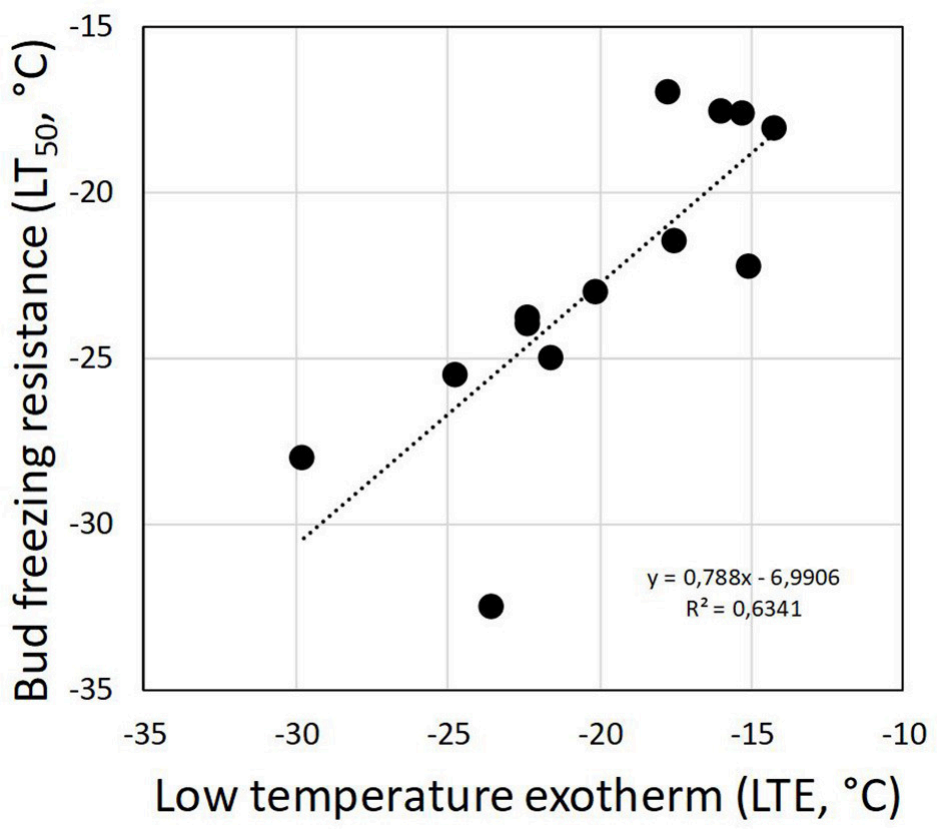
Correlation between the detected low-temperature exotherms (LTEs, °C) in temporary supercooled buds and measured FR (LT_50_, °C).

Although DTA does not allow one to determine the location where ice forms in tissues, it is very sensitive and can detect small freezing events. When properly configured, it also allows one to measure a considerable number of buds as replicates at the same time. HTEs in buds of the 37 species were detected at a mean freezing temperature of −1.6 ± 0.9°C (SD). The temperature of the HTE varied slightly among the tested species (Table 1). Collectively, HTEs occurred between −0.5°C in *Fagus sylvatica* and −3.2°C in *Celtis occidentalis*. In the majority of the species (73%), however, water in stem tissues froze extracellularly between −1.0 and −2.1°C. Buds that exhibited an absence of any supercooling exhibited a mean HTE that occurred at a slightly but significantly higher freezing temperature (−1.2°C) than in the other bud types. Buds that exhibited supercooling, but no LTE, had a mean HTE of −1.5°C; meanwhile, while buds that exhibited temporary supercooling and a LTE had a mean HTE of −1.7°C. A distinct LTE could be detected by DTA analysis during controlled freezing in 14 (38%) of the examined species (Figure 2). These results corresponded with the results obtained by IDTA fully (Table 1). More specifically, intracellular freezing of the bud tissue could be visualized by IDTA at a temperature similar to the LTE detected during DTA. Mean values of the recorded LTEs ranged between −14.2°C (*Aesculus hippocastanum*) and −29.8°C (*Ilex aquifolium*) with an overall mean of −20.5 ± 4.6 SD°C. Mean ± SD of FR (LT_50_) was −22.8 ± 4.4°C, and LTE temperatures correlated well with the FR (LT_50_) of buds (Figure 3).

**Figure 4:**
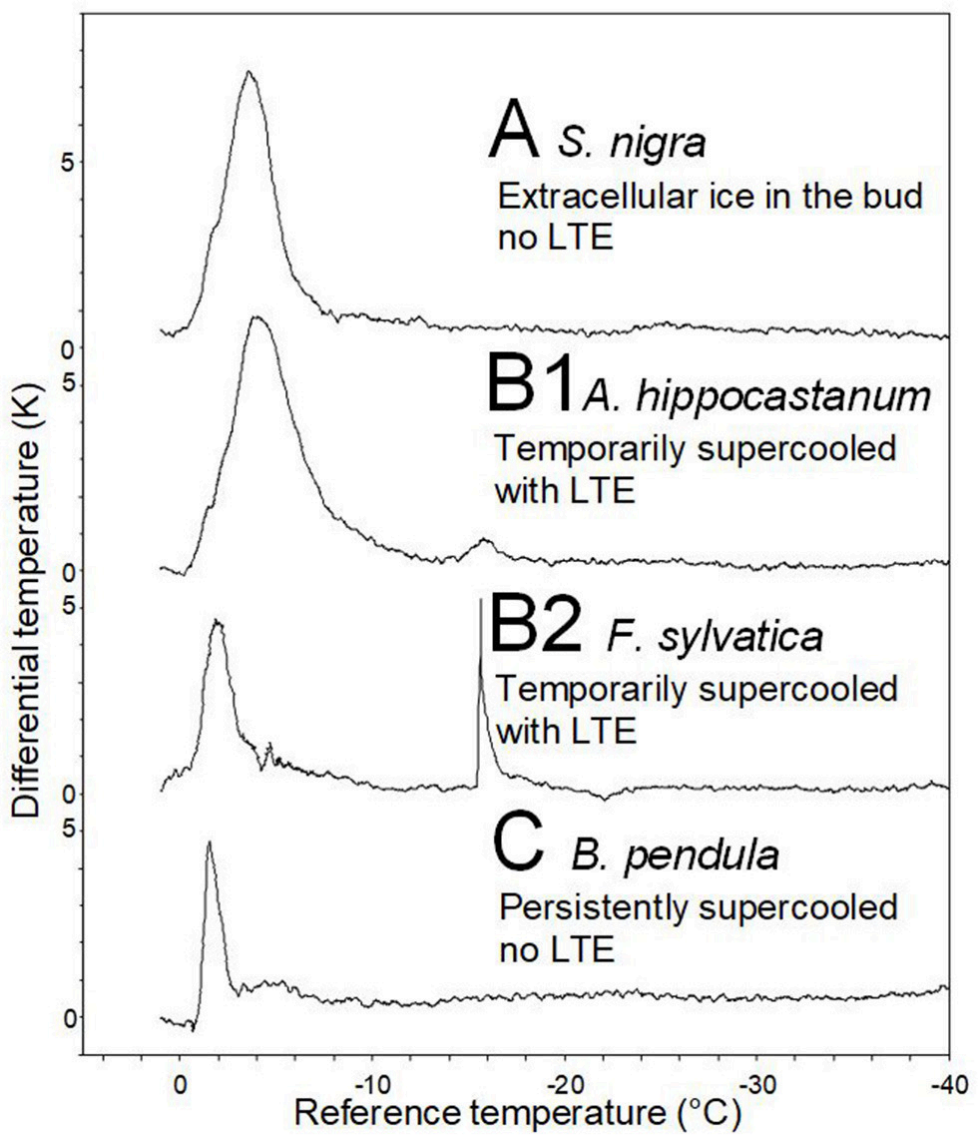
Representative differential thermal analysis plots (DTA-plots) obtained from buds during controlled freezing treatments (cooling rate: 5 K/h). Three freezing patterns could be distinguished by the DTA-plots: **(A)** *S. nigra*, whose buds freeze extracellularly during HTE, **(B1)** *A. hippocastanum* and **(B2)** *F. sylvatica*, whose buds supercool temporary but freeze intracellularly at some critical threshold temperature as indicated by the low temperature exotherm (LTE), and **(C)** *B. pendula*, where buds also supercool but do not freeze intracellularly (No LTE).

Representative DTA-plots that depict the three different freezing pattern types are shown in Figure 4. Buds of *S. nigra* freeze extracellularly during the occurrence of the HTE. The HTE in buds that did not supercool was typically a distinct, long-lasting exotherm in the DTA-plot. Buds of *A. hippocastanum* and *F. sylvatica* supercool but exhibit an LTE when the temperature falls below their ability to supercool. The HTE in supercooled buds that exhibit an LTE is distinct and can be similar (*A. hippocastanum*) or much shorter (*F. sylvatica*) than the HTE in buds that freeze extracellularly and do not supercool. LTEs in these buds were often short and pinnacle (*F. sylvatica*) freezing exotherms in the DTA but could also appear as a small hillock (*A. hippocastanum*), the latter indicating a slower freezing process. Lastly, buds that supercool but do not exhibit a distinct LTE are exemplified by *B. pendula*. The DTA-plot in these types of buds is characterized by a short but distinct HTE and the absence of any LTE.

**Figure 5:**
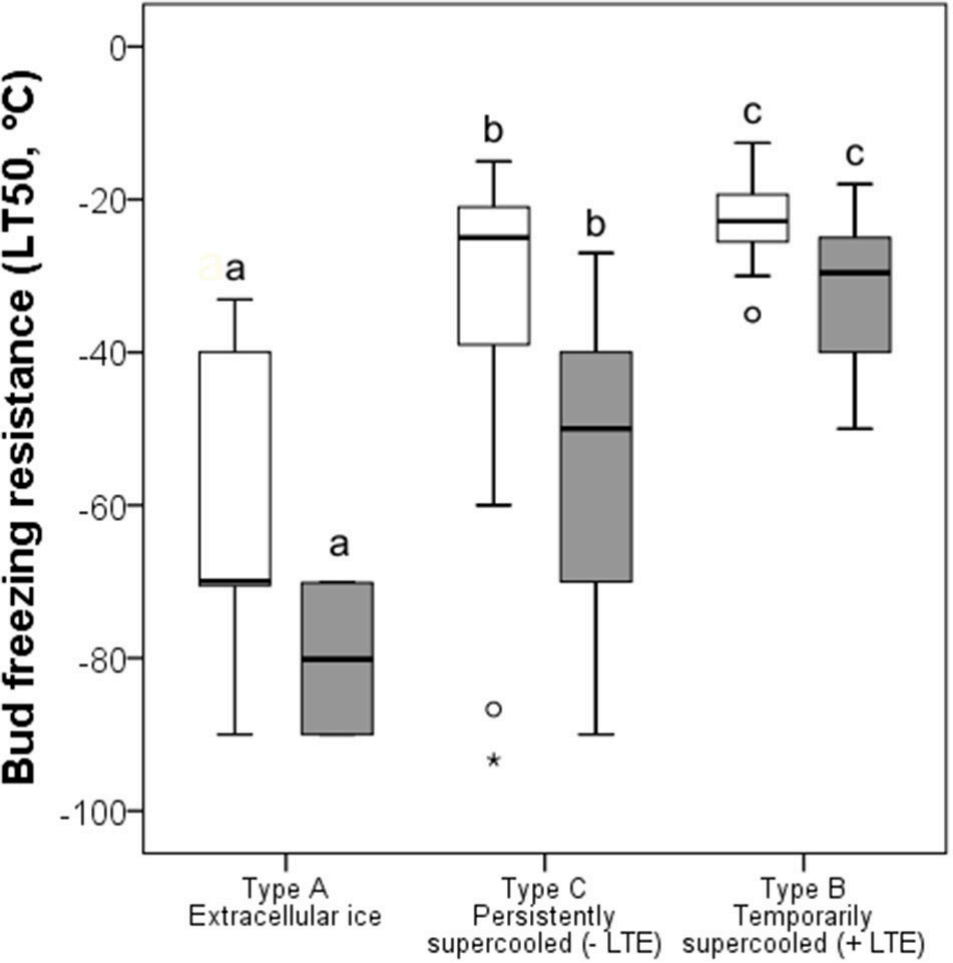
Comparison of bud FR (LT_50_, °C) determined (white bars) in the current study and (gray bars) by other authors (see Table 1) grouped by contrasting frost survival mechanisms: *Type A* extracellular freezing (ice tolerant), *Type B* temporarily supercooled with the occurrence of intracellular freezing as evidenced by an LTE, and *Type C* persistently supercooled without lethal intracellular freezing (no LTE). The box plots indicate the median (= second quartile; line inside the box) and extend from the first to the third quartile. The whiskers indicate, at maximum, the 1.5-fold interquartile range. Outliers are shown as dots, extreme outliers as stars. The significance of difference between bud FR of different bud types is indicated by different letters (*P* < 0.05).

Under the winter conditions of 2015/16, buds did not frost harden to the midwinter maximum reported in the literature, but differences in FR of functional groups was similar (Figure 5). The most frost hardy, with a mean midwinter LT_50_ of −61.8°C, were buds that exhibited ice tolerance. Temporarily supercooled buds that exhibited an LTE, with some exceptions, were the least frost hardy, exhibiting a mean LT_50_ of −22.8°C. Buds that supercooled but that did not exhibit an LTE exhibited an intermediate level of FR (LT_50_ −31.6°C). Some species within this group, however, were very frost susceptible, such as *Euonymus europaeus* with an LT_50_ of −19.0°C, while others, such as *B. pendula*, exhibited a level of FR similar to buds that froze extracellularly and did not supercool or exhibit an LTE.

**Figure 6:**
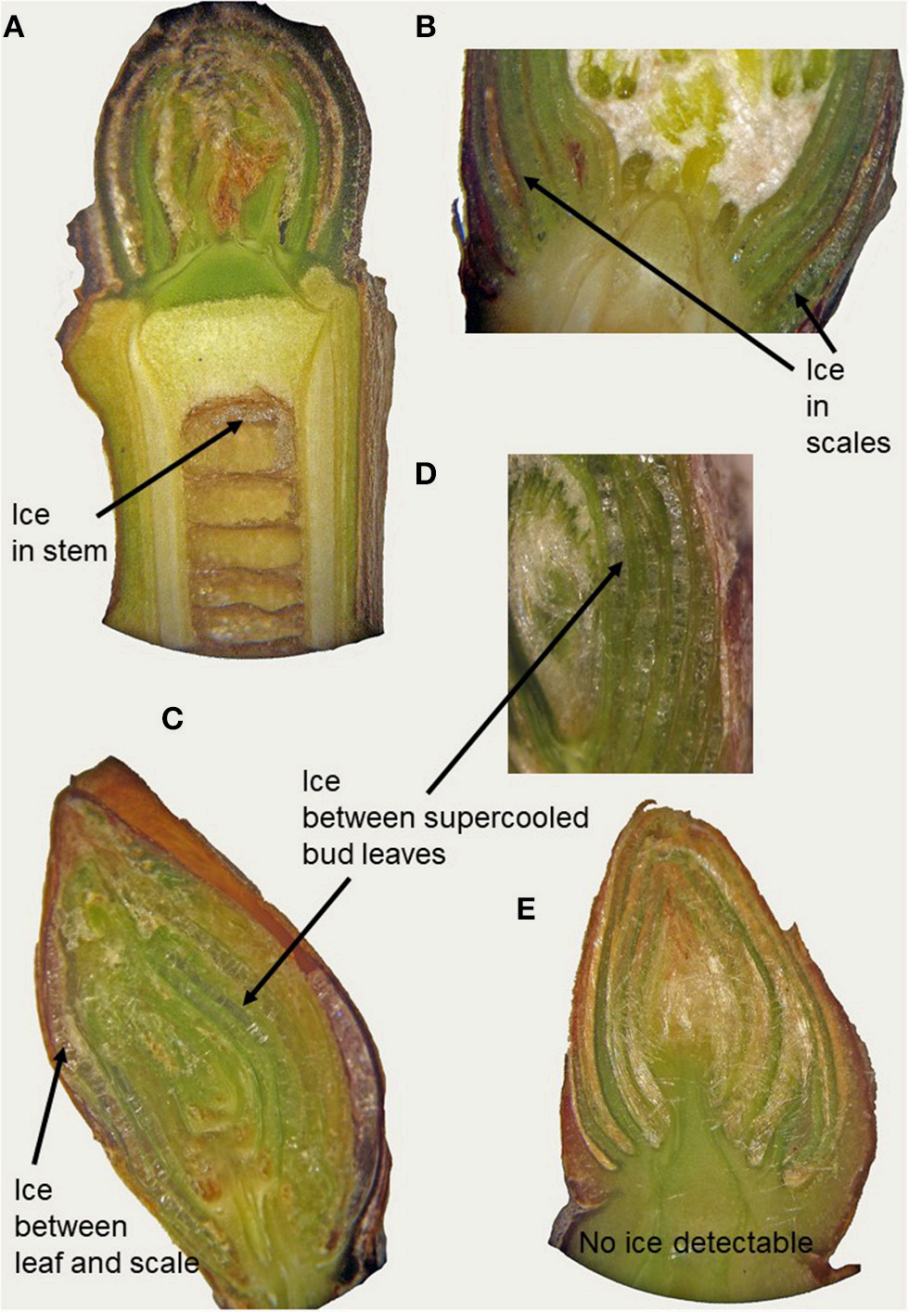
By cryo-microscopy extra organ ice masses could be detected in different locations in buds sectioned longitudinally in the frozen state (−10°C). Ice was found either in **(A)** the subtending stem (*J. regia*), in **(B)** the bud scales (*A. hippocastanum*) or inside the bud around the premature leaves as in **(C)** *B. pendula* and in **(D)** *O. carpinifolia* or **(E)** no ice could be found inside the bud or in tissues in close vicinity to the bud.

**Figure 7:**
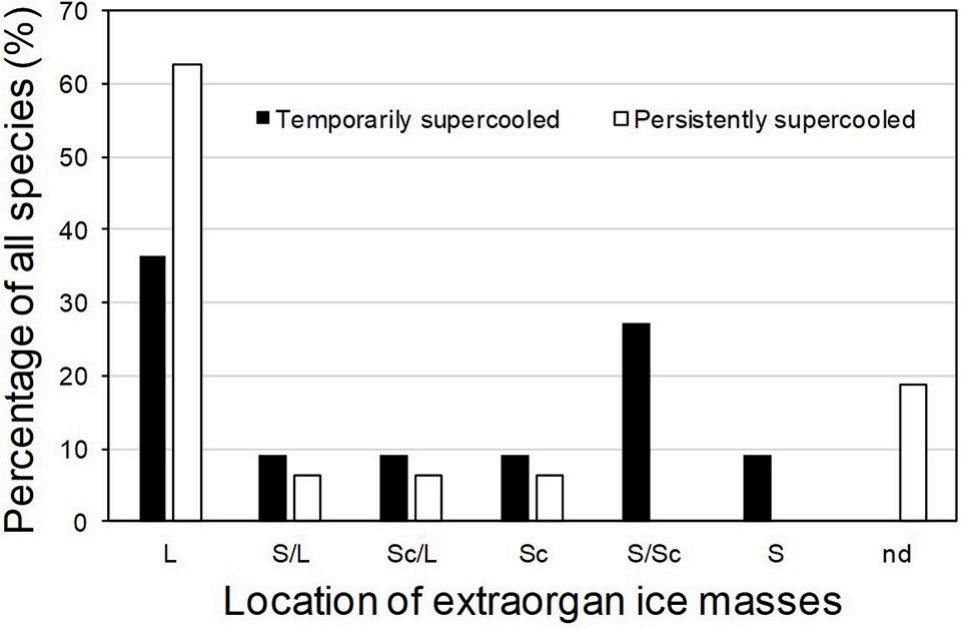
In supercooled buds, translocated ice masses can be found either in (S) the adjoining stem, in (Sc) the bud scales or inside (L) the bud around the premature leaves. In some buds, (nd) no large ice masses could be found inside or in close vicinity to the bud. Percentage of all species using the respective locations for translocated ice masses are given for (■) temporarily supercooled and (□) persistently supercooled buds.

In supercooling buds, formation of extraorgan ice masses was monitored by cryo-microscopy (Table 1). At −10°C in the majority of the investigated species, extraorgan ice masses could be detected either in the adjoining stem (Figure 6A), in the bud scales (Figure 6B) or inside the bud around the premature leaves (Figures 6C,D). The exceptions were four species with persistently supercooling buds (*Castanea sativa, L. anagryoides, Quercus rubra, Salix caprea*), where no large ice masses could be found inside or in close vicinity to the bud (Figure 6E). In 50% of all tested species, translocated ice masses formed exclusively around the premature leaves inside the bud. While in DTA, no freezing exotherms were detectable during formation of translocated ice between the premature leaves, in some species in IDTA occasionally exiguous freezing processes were recorded (e.g., *A. alnobetula*: Neuner et al., 2019). In temporarily supercooled buds, no preferential location of ice masses could be found (Figure 7). In persistently supercooled buds, except for the species lacking ice masses, ice crystals mostly could be found close to premature leaves, but in *Populus tremula* and *Corylus avellana* they could additionally be found in the scales and in *Viburnum lantana* additionally in the stem—and only in *Sorbus aucuparia* was ice exclusively seen in the bud scales.

## Discussion

### Frost Survival Mechanisms of Vegetative Buds

#### Extracellular Freezing of Buds (Type A)

Vegetative buds that freeze extracellularly, i.e., Type A buds, have not been experimentally proven until now in angiosperms (Sakai and Larcher, 1987) but have been found in *S. nigra* and *E. rhamnoides*. Interestingly, reproductive buds in *Sambucus racemosa*, in contrast to the many reproductive buds of angiosperm woody species (Ashworth, 1982, 1990; Quamme, 1995), also did not exhibit an LTE (Ishikawa and Sakai, 1982); this suggests that another FR mechanism exists other than deep supercooling. With respect to conifers, the results obtained in *P. cembra* and *P. sylvestris* corroborate earlier findings that buds of pines survive by extracellular freezing (Sakai and Eiga, 1985; Price et al., 1997; Ide et al., 1998). Buds that only exhibit extracellular freezing with an absence of any supercooling were the most frost hardy, corroborating earlier reports on the FR of *P. cembra* and *P. sylvestris* that exhibited a maximum FR at midwinter of −90°C (Sakai and Okada, 1971). Opposed to other conifers that have buds that supercool (Quamme, 1995), in pines, ice forms in the buds at the same time when ice forms in subtending stem tissues, and no supercooling occurs (Ide et al., 1998). In contrast to other *Pinaceae*, such as *Abies* (Sakai, 1978; Ide et al., 1998) and *Picea* (Sakai, 1979; Kuprian et al., 2017, 2018), pines also do not have crown tissue at the base of the bud, which serves as an ice barrier that keeps the bud free of ice. Similar to pines, the two angiosperms examined in the present study, *S. nigra* and *E. rhamnoides*, also do not have structural barriers that impede ice propagation from the stem into the bud. Additionally, a barrier against extrinsic ice nucleation from the bud surface does not appear to exist in buds that exhibit extracellular freezing. *S. nigra* buds for instance differ from other buds as they lack a compact, tight coverage of buds by layers of bud scales. In supercooling buds, an impermeable ice barrier can be brought about by a sophisticated bud scale architecture (*P. abies*, Kuprian et al., 2017) or, as only recently reported, by surface impregnation with lipophilic substances (Neuner et al., 2019).

Ice nucleation was triggered in the stem by the use of ice-nucleation-active (INA) bacteria. It is important to artificially nucleate the twig samples, as otherwise ice nucleation would occur at much lower freezing temperatures than those found in natural settings (data not shown). This has been recently demonstrated in *P. abies*, where ice nucleation in detached twigs does not occur at temperatures warmer than −8.4°C in the absence of INA bacteria. However, when INA bacteria are used, ice nucleation is triggered around −2.9°C (Kuprian et al., 2017). The temperature range (−0.5 to −3.2°C), wherein HTEs occurred in the detached twigs of the 37 species examined in the present study, was similar to the range of temperatures within which ice nucleation has been reported to occur in intact woody plants in nature, i.e., > −3.4°C (Beck et al., 1982; Ashworth and Davis, 1986; Mayr et al., 2006; Buchner and Neuner, 2011; Pramsohler et al., 2012). In nature, ice nucleation in stem tissues at relatively warm sub-zero temperatures appears to be advantageous. The water potential gradient from the liquid cellular water to the surrounding extracellular ice is temperature-dependent and increases significantly with decreasing temperature. Artificial supercooling could expose cells to a steep water potential gradient when ice forms that could potentially result in injury that otherwise would not be seen.

#### Buds Surviving Free of Ice

In the majority (89.2%) of the investigated species, buds remained free of ice during and after the HTE. Deviating from earlier expectations (Sakai and Larcher, 1987), most vegetative buds of angiosperms survive freezing temperatures free of ice, and this appears to be the general rule rather than the exception. Only a few reports have suggested this type of freezing response in vegetative buds of angiosperms, including *P. syriaca* (Rajashekar and Burke, 1978), *A. japonicum* (Ishikawa et al., 1997), and *M. domestica* (Pramsohler and Neuner, 2013). In contrast, this type of freezing response is generally accepted to be the major mechanism in most vegetative buds of conifers, with the exception of pines (Zwiazek et al., 2001).

Maintenance of an ice-free bud during and after the HTE requires the presence of external and internal ice barriers. Once ice has formed anywhere in the plant (in nature > −3.4°C) and comes into contact with xylem-conducting elements, it usually spreads rapidly (at high rates of up to 27 cm.s^−1^) into all plant parts that are colder than 0°C. Furthermore, the ice is able to spread into areas that are not protected by an ice barrier (Wisniewski et al., 1997; Hacker and Neuner, 2007, 2008; Hacker et al., 2008). Therefore, an internal ice barrier that prevents the spread of ice into a bud is necessary. By IDTA, for all supercooling buds it could be shown that ice propagation from the frozen subtending stem into the bud is prevented by an internal ice barrier. Nevertheless, the bud also needs to be protected from extrinsic nucleation events that occur on the bud surface. These structural requirements may be met by a unique water/ice proof bud architecture where the scales and cuticle play an important role as an impermeable barrier (Kuprian et al., 2017) or by surface impregnation with lipophilic substances (triterpenoids and flavonoid aglycones: Neuner et al., 2019). Similarly, in some species, premature leaves are spaced by a dense trichome felt that we suggest might have a comparable function.

In addition to the presence of ice barriers, maintenance of the supercooled state requires regulatory mechanisms that are involved in the freezing response, including potential biochemical components that have supercooling stabilizing activity, ice nucleation activity and/or antifreeze activity (Ishikawa et al., 2009). The biochemical mechanisms involved in freeze regulation that likely determine freezing response remain unclear (Ishikawa et al., 2009, 2015; Wisniewski et al., 2009, 2014; Kishimoto et al., 2014).

Freeze dehydration of the supercooled bud could also play a role in maintaining the supercooled state (Ide et al., 1998); however, desiccation alone did not increase the supercooling capacity of buds in *P. abies* (Kuprian et al., 2018). Still, the freezing of apoplastic water in the stem and subtending bud scales, while buds remain ice-free, produces a steep water potential gradient that must be managed. Typically, water migrates from the bud across the barrier tissue to the site of the ice, which has been labeled as extraorgan freezing (Sakai, 1982). Different degrees of freeze dehydration are also the basis of the postulated bud survival typology described by Sakai and Larcher (1987). Recently, temperature-dependent freeze dehydration of supercooled buds was demonstrated in *P. abies* (*Type III*) (Kuprian et al., 2017). Strikingly, the extent of freeze dehydration, as indicated by measuring the water potential of buds, was not much different than that reported for buds of *M. domestica* (Pramsohler and Neuner, 2013), which are *Type II* buds. These results suggest that differences in the extent of freeze dehydration may not explain the differences that exist between the low-temperature survival of *Type II* and *III* buds. Our typology does not use the amount of freeze dehydration for classification.

Extraorgan freezing depends on the presence of ice sinks in the subtending stem, at the base of the bud scales or, as has been recently shown, between the premature leaves inside the bud (Neuner et al., 2019). In *P. abies* (Kuprian et al., 2017), translocated water freezes and forms large ice masses in voids in the pith tissue of the subtending stem. By stereo-light microscopy and cryo-SEM it could be shown that in *Larix kaempferi* large extraorgan ice masses form in the subtending stem in the space below the crown tissue and within basal areas of scales (Endoh et al., 2009), which is a typical pattern of extraorgan freezing in conifers (Sakai, 1979, 1982). Freezing exotherms that are provoked by formation of translocated ice are usually not detectable by DTA, as the freezing process may be too slow. In some species, such as *A. alnobetula*, exiguous freezing signals from ice formation between the leaves could be seen in IDTA (Neuner et al., 2019). However, as in *P. abies*, no freezing exotherm may be recorded by IDTA (Kuprian et al., 2017). Supercooled buds of angiosperms have also been shown to accumulate large ice masses within the basal parts of bud scales and the upper part of the immature pith of the subtending stem, as has been reported for *A. japonicum*, (Ishikawa et al., 1997) and *Acer pseudoplatanus* (Dereuddre, 1979). The ice masses create cavities that can be seen as such when the stem and bud tissues thaw. Interestingly, our results suggest that in more than 50% of the investigated species, and in most of the persistently supercooling buds, translocated ice masses harmlessly form between the premature leaves, such as what was recently reported for *A. alnobetula* (Neuner et al., 2019). While in stems and bud scales the growing ice masses disrupt the tissue, this is not the case in the latter, which may be advantageous.

#### Temporarily vs. Persistently Supercooled Buds

Two different frost survival mechanisms could be distinguished in buds that remain free of ice during and after the HTE. In the first mechanism, buds continued to supercool but were killed by an intracellular freezing event initiated within the bud when the temperature fell below the ability of the buds to maintain supercooling. This freezing event was detectable by an LTE and such buds were termed Type B buds, which corresponds to the *Type III* buds of Sakai and Larcher (1987). As supercooling in Type B buds can suddenly break down, we term it ***temporarily supercooled***. In the second mechanism, the buds remain supercooled; however, no further freezing occurs. This type of response represents *Type I* or *II* buds (Sakai and Larcher, 1987), where *Type I* buds are thought to be fully dehydration-tolerant with unlimited FR and *Type II* buds can be intensively dehydrated, although they are not dehydration-tolerant with limited FR (−35 to −50°C). Freeze dehydration was not examined in the current study, and the level of FR measured in winter 2015/16 may not be the species-specific maximum. Therefore, a clear assignment of the buds examined in this current study as *Type I* or *II* buds is not possible. Additionally, freeze dehydration of many buds that remain supercooled without a detectable LTE is generally difficult to assess. Therefore, we suggestively term this group of buds Type C, or ***persistently supercooled***.

#### Temporarily Supercooled Buds (Type B)

In 14 of the examined species, the ice-free, supercooled bud cells were observed to freeze intracellularly in a sudden event, detectable as an LTE. This occurred at a temperature below a certain threshold freezing temperature that corresponded to the frost killing temperature. Similar to the observations on Norway spruce buds (Kuprian et al., 2017), ice never penetrated the ice barrier between the adjoining stem and the bud, but lethal freezing was in all temporarily supercooling species initiated by an independent, separate ice nucleation event inside the bud tissue. Once ice had nucleated, the bud tissue froze within seconds. The FR of these temporarily supercooled buds was much less, relative to the other bud types. The LT_50_ in these species ranged from −17.0°C in *Crataegus monogyna* to −32.5°C in *A. platanoides* (mean −22.8°C). Other species with Type B buds have been reported to attain similar moderate levels of midwinter FR, such as *A. pseudoplatanus*, with a mean FR of −21°C (Larcher, 1985) and *Juglans regia*, with a mean FR of −18.5°C (Charrier et al., 2013). The maximum observed FR of −32.5°C corresponds to the FR limit (−25 to −30°C) indicated by Sakai and Larcher (1987) for this type of buds. Other reports on maximum midwinter FR obtained for temporarily supercooling vegetative buds of angiosperms, however, suggest that in extreme survival of freezing temperatures for the majority of down to −40°C is possible, exceptionally even as low as −50°C (*P. abies*: Beuker et al., 1998). For example, maximum midwinter FR of *F. sylvatica* buds was reported to reach −40°C (Lenz et al., 2013, 2016; Kreyling et al., 2014), which is similar to the level (−40°C) reported for *A. hippocastanum* (Sakai and Larcher, 1987). Nevertheless, the limited maximum FR of temporarily supercooling buds likely limits the northern distribution of species that employ this functional frost survival strategy. A similar conclusion was drawn by Quamme (1995) on the basis of knowledge at that time for deep supercooling buds of conifers and reproductive buds of angiosperms.

#### Persistently Supercooled Buds (Type C)

In 51.4% of the investigated species, vegetative buds remained ice-free during and after the HTE and did not exhibit any further freezing event (LTE) that was associated with frost injury. Such freezing behavior is also known for flower buds of angiosperms that do not exhibit an LTE (e.g., apple: Quamme, 1991). FR of Type C buds was intermediate. Some species were rather frost susceptible, while others were among the most frost hardy. For example, overwintering buds of *B. pendula* have been reported to survive liquid hydrogen temperatures (−253°C) if properly acclimated (Sakai and Larcher, 1987). It was suggested that Type C buds become freeze-dehydrated to such an extent that no freezable water remains that could be involved in producing an LTE. This could suggest that another injurious process, other than intracellular freezing, occurs in Type C buds (Sakai and Larcher, 1987). However, as when the intracellular freezing process is insignificantly below the resolution limit of DTA and IDTA, intracellular freezing as a cause of frost damage to Type C buds cannot be completely excluded. Previous DTA studies showed that experimental settings such as slow cooling rates (Endoh et al., 2009) or prolonged exposure to freezing conditions that promote dehydration (Sakai, 1979) can lead to failure of detection of an LTE in buds. Similarly, xylem ray parenchyma cells of boreal trees showed deep supercooling in a cryo-SEM study, while DTA did not provide an LTE (Kuroda et al., 2003). Further studies are needed to clarify how these persistently supercooled buds are killed.

#### Variability of Midwinter Bud FR

In winter 2015/16—compared to earlier reports—the tested species were less frost hardy, which can be addressed to milder site conditions and the relatively moderate winter temperatures. Midwinter bud FR is attained after a long frost hardening period that is initiated in autumn by decreasing photoperiod and colder temperatures (Horvath et al., 2003). In the dormant state, actual bud FR can largely fluctuate in response to abrupt temperature variations (Hänninen, 2016; Lenz et al., 2016; Vitra et al., 2017). For instance, in Norway spruce inter-annual differences in winter temperatures caused different midwinter bud FR between years and sudden low temperature exposure enforced immediate additional frost hardening in the buds (Beuker et al., 1998).

Despite exposure to similar environmental conditions, midwinter bud FR differed intra-specifically between −17.0 and −90.0°C. The extreme differences between species can only partly be explained by different frost survival mechanisms. The results clearly point out that the temporarily supercooled buds have a limited midwinter bud FR for most species down to −40°C, or at the extreme −50°C. Extracellularly freezing buds generally belong to the frost hardiest group (−90°C). Strikingly, within the species that have persistently supercooled buds, a similar heterogeneity with respect to FR was observed (−19.0 to −90.0°C) as between all species. This may be indicative for currently unknown mechanistic differences within this frost survival type. The extreme intra-specific differences in midwinter FR developed under similar environmental conditions point out that besides winter temperature, there is also a strong functional and genetic component effectively leaving species with dramatically different safety margins from frost damage.

## Author Contributions

GN designed the research, wrote the manuscript and interpreted the results. DK, JI, and KM were responsible for the performance of the research, data analysis, collection and interpretation.

### Conflict of Interest Statement

The authors declare that the research was conducted in the absence of any commercial or financial relationships that could be construed as a potential conflict of interest.
