# Supplementary material for: Frost Survival Mechanism of Vegetative Buds in Temperate Trees: Deep Supercooling and Extraorgan Freezing vs. Ice Tolerance
Source: Front Plant Sci. 2019 May 9;10:537. doi: 10.3389/fpls.2019.00537 (PMC6521125; doi:10.3389/fpls.2019.00537)
Supplement: Supplementary file 1 [file Table_1.pdf]

Supplementary table 1. List of the investigated woody plant species: sampling date (date of freezing resistance measurements are in *italics*), sampling site, and altitude (meters above sea level) of the collection site, and an indication of whether the sampled plants are (o) ornamentals or (n) native species in the study area.

Plant species	Sampling date 2016	Sampling site	Altitude (m a.s.l.)	Native (n)/ Ornamental (o)
Acer platanoides L.	2.2./*22.2.*	47°15'21.4"N 11°21'37.6"E	580	n
Acer pseudoplatanus L.	10.1. /*22.2.*	47°15'21.4"N 11°21'37.6"E	580	n
Aesculus hippocastanum L.	26.1. /*22.2.*	47°16'05.7"N 11°22'43.8"E	611	o
Alnus incana (L.) Moench	2.2. /*22.2.*	47°16'05.7"N 11°22'43.8"E	611	n
Alnus alnobetula (Ehrh.) K.Koch	27.1. /*22.2.*	47°16'05.7"N 11°22'43.8"E	611	n
Betula pendula Roth	3.1./ 7.1/*22.2.*	47°16'05.7"N 11°22'43.8"E /47°15'29.4"N 11°21'54.9"E	611/580	n
Carpinus betulus L.	14.1. /*22.2.*	47°16'05.7"N 11°22'43.8"E	611	n
Castanea sativa Mill.	*24.2.*	47°16'03.56"N 11°22'43.99"E	618	o
Catalpa bignoniodes Walter	*25.2.*	47°16'15.59N 11°11'17.40"E	678	o
Celtis occidentalis L.	*29.2.*	47°16'02.18"N 11°22'43.08"E	616	o
Cornus mas L.	*25.2.*	47°16'15.28"N 11°11'17.13"E	679	n
Corylus avellana L.	4.1./ 7.1. /*22.2.*	47°16'05.7"N 11°22'43.8"E /47°15'21.4"N 11°21'37.6"E	611/580	n
Crataegus monogyna Jacq.	*25.2.*	47°16'15.89"N 11°11'18.72"E	675	n
Elaeagnus rhamnoides (L.) A. Nelson	*24.2.*	47°16'04.84"N 11°22'47.89"E	617	n
Euonymus europaeus L.	*29.2.*	47°16'03.21"N 11°11'47.64"E	609	n
Fagus sylvatica L.	11.1. /*22.2.*	47°16'05.7"N 11°22'43.8"E	611	n
Ilex aquifolium L.	*24.2.*	47°16'03.20"N 11°22'48.04"E	610	o
Juglans regia L.	5.1. /*22.2.*	47°16'05.7"N 11°22'43.8"E	611	o
Laburnum anagyroides Medik.	*25.2.*	47°16'16.75"N 11°11'17.71"E	672	o
Liriodendron tulipifera L.	*24.2.*	47°16'04.11"N 11°22'44.67"E	615	o
Morus nigra L.	*25.2.*	47°16'16.50"N 11°11'17.82"E	675	o
Ostrya carpinifolia Scop.	*24.2.*	47°16'02.97"N 11°22'43.88"E	613	n
Picea abies (L.) H.Karst.	11.1. /*22.2.*	47°16'05.7"N 11°22'43.8"E	611	n
Pinus cembra L.	25.1. /*22.2.*	47°12'25.8"N 11°27'09.2"E	1980	n
Pinus sylvestris L.	29.1. /*22.2.*	47°16'21.3"N 11°22'50.7"E	720	n
Platanus x hispanica Münchh.	*24.2.*	47°16'02.86"N 11°22'46.12"E	609	o
Populus tremula L.	*25.2.*	47°16'15.29"N 11°11'21.50"E	675	n
Prunus avium L.	*29.2.*	47°16'16.36"N 11°11'18.62"E	673	n
Quercus rubra L.	*24.2.*	47°16'03.88"N 11°22'43.25"E	615	o
Rhododendron ferrugineum L.	25.1. /*22.2.*	47°12'25.8"N 11°27'09.2"E	1980	n
Salix caprea L.	3.2. /*22.2.*	47°16'05.7"N 11°22'43.8"E	611	n
Salix helvetica Vill.	*29.2.*	47°16'04.83"N 11°22'51.00"E	622	n
Sambucus nigra L.	6.1./7.1. /*22.2.*	47°16'05.7"N 11°22'43.8"E /47°15'29.4"N 11°21'54.9"E	611/580	n
Sorbus aucuparia L.	17.1. /*22.2.*	47°17'13.4"N 11°31'26.1"E	605	n
Tilia cordata Mill.	17.1. /*22.2.*	47°15'21.4"N 11°21'37.6"E	580	n
Ulmus glabra Huds.	*2.3.*	47°16'1.88"N 11°15'41.10"E	588	n
Viburnum lantana L.	*24.2.*	47°16'03.80"N 11°22'41.88"E	616	n
