# Supplementary material for: Frost Survival Mechanism of Vegetative Buds in Temperate Trees: Deep Supercooling and Extraorgan Freezing vs. Ice Tolerance
Source: Front Plant Sci. 2019 May 9;10:537. doi: 10.3389/fpls.2019.00537 (PMC6521125; doi:10.3389/fpls.2019.00537)
Supplement: Supplementary file 6 [file Image_1.pdf]

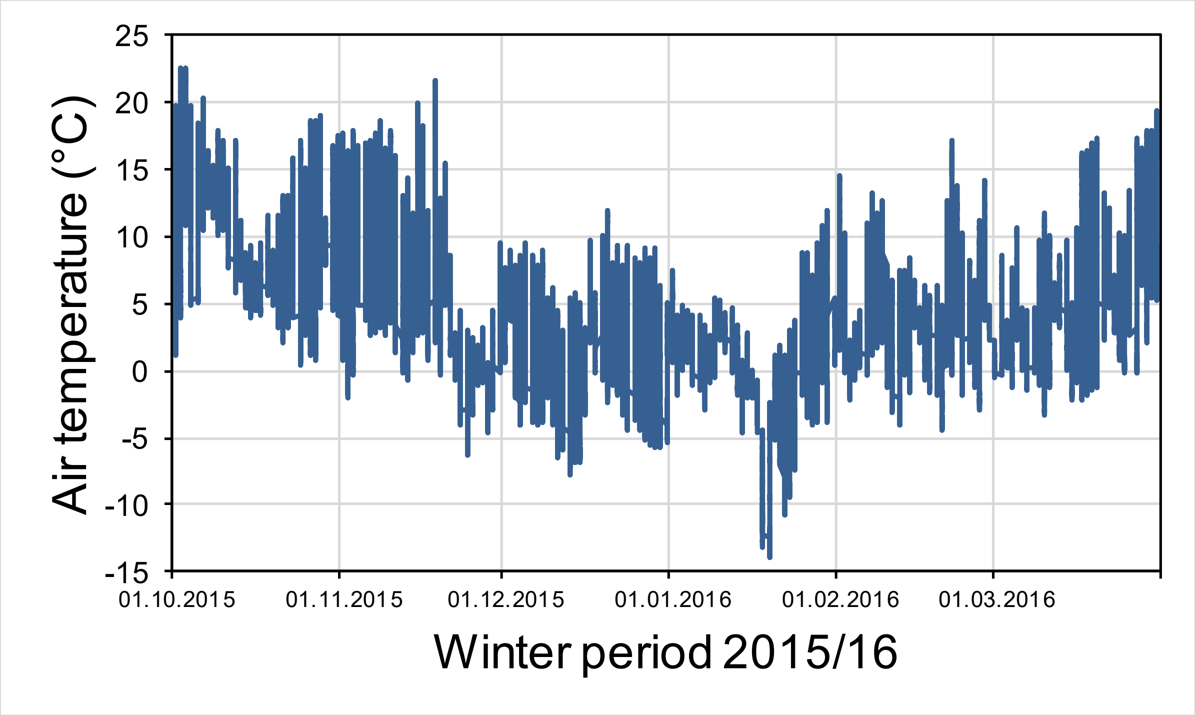

Supplementary Figure 1. Air temperatures recorded at Innsbruck airport from October 2015 till March 2016 (data supplied by Lawinenwarndienst Tirol).

frontiers
