# Supplementary material for: Frost Survival Mechanism of Vegetative Buds in Temperate Trees: Deep Supercooling and Extraorgan Freezing vs. Ice Tolerance
Source: Front Plant Sci. 2019 May 9;10:537. doi: 10.3389/fpls.2019.00537 (PMC6521125; doi:10.3389/fpls.2019.00537)
Supplement: Supplementary file 7 [file Image_2.pdf]

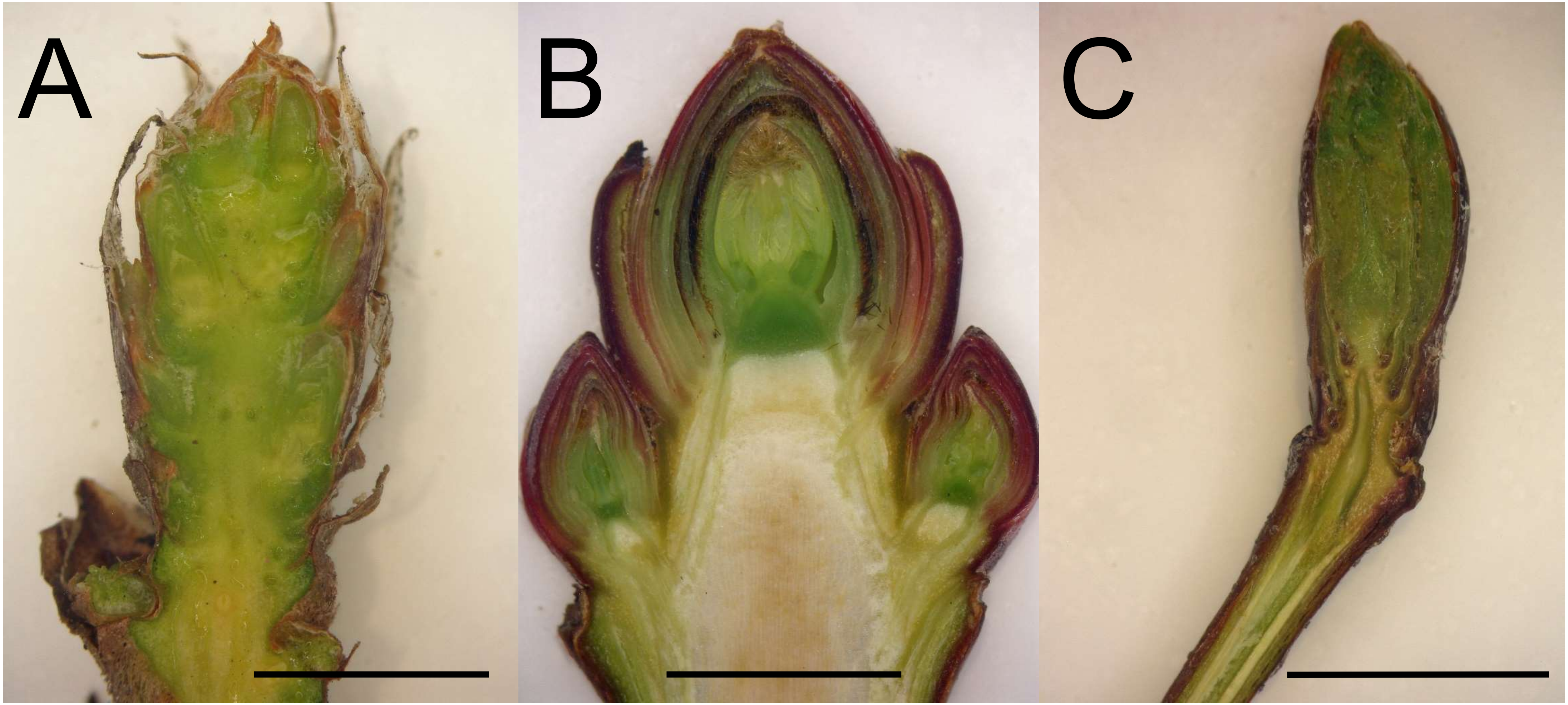

Supplementary figure 2. Digital colour images of longitudinally sectioned shoots bearing vegetative buds of (A) *Pinus cembra* (Type A – extracellular freezing), (B) *Acer platanoides* (Type B – temporarily supercooled) and (C) *Betula pendula* (Type C – persistently supercooled). Horizontal black bars are 5 mm.

frontiers
